# Forensic Medicine and Toxicology

**Published:** 1899-03

**Authors:** 


**Forensic Medicine and Toxicology.** By J. DIXON MANN, M.D. Second Edition. Pp. xiv., 683. London: Charles Griffin and Company, Limited. 1898.

In March, 1894, we noticed the first edition of this work, and considered that it would prove useful for students to peruse before an examination, owing to the condensation of its matter, and help the general practitioner who has no time to refer to the larger manuals. In this second edition improvements have been introduced in many parts. Cases are more frequently given and at more length. Some useful paragraphs have been added in the section dealing with sudden death from natural causes. Under the head of "Life Assurance," something, we think, ought to have been given as the probable duration of life in those suffering from organic diseases: a rather difficult subject, no doubt, but one which is only slightly treated even by the authorities on general disease, to whom we are referred by Dr. Mann. We notice the absence of any account of gunshot wounds caused by modern weapons of precision and the new bullets—for some strange accidents have been caused by them, and suicides effected by their use. Under "Heat-Stroke" there is no mention of how frequently insanity of a dangerous and incurable form follows on an attack. Amongst the poisons, acetylene, now coming so much into use, is noticed, as are also some of the aniline group and poisonous foods.

